# Immunoinformatics approach of epitope prediction for SARS-CoV-2

**DOI:** 10.1186/s43141-022-00344-1

**Published:** 2022-04-20

**Authors:** Nourelislam Awad, Rania Hassan Mohamed, Nehal I. Ghoneim, Ahmed O. Elmehrath, Nagwa El-Badri

**Affiliations:** 1 Center of Excellence for Stem Cells and Regenerative Medicine (CESC), Zewail City of Science and Technology, Giza, Egypt; 2 Center of Informatics Sciences, Nile University, Giza, Egypt; 3 Department of Biochemistry, Faculty of Science, Ain Shams University, Cairo, Egypt; 4 Faculty of Medicine, Cairo University, Cairo, Egypt

**Keywords:** SARS-CoV-2, MHC class I epitopes, ORF1ab protein, Spike protein, Immunoinformatics

## Abstract

**Background:**

The novel coronavirus (SARS-CoV-2) caused lethal infections worldwide during an unprecedented pandemic. Identification of the candidate viral epitopes is the first step in the design of vaccines against the viral infection. Several immunoinformatic approaches were employed to identify the SARS-CoV-2 epitopes that bind specifically with the major histocompatibility molecules class I (MHC-I). We utilized immunoinformatic tools to analyze the whole viral protein sequences, to identify the SARS-CoV-2 epitopes responsible for binding to the most frequent human leukocyte antigen (HLA) alleles in the Egyptian population. These alleles were also found with high frequency in other populations worldwide.

**Results:**

Molecular docking approach showed that using the co-crystallized MHC-I and T cell receptor (TCR) instead of using MHC-I structure only, significantly enhanced docking scores and stabilized the conformation, as well as the binding affinity of the identified SARS-CoV-2 epitopes. Our approach directly predicts 7 potential vaccine subunits from the available SARS-CoV-2 spike and ORF1ab protein sequence. This prediction has been confirmed by published experimentally validated and in silico predicted spike epitope. On the other hand, we predicted novel epitopes (RDLPQGFSA and FCLEASFNY) showing high docking scores and antigenicity response with both MHC-I and TCR. Moreover, antigenicity, allergenicity, toxicity, and physicochemical properties of the predicted SARS-CoV-2 epitopes were evaluated via state-of-the-art bioinformatic approaches, showing high efficacy of the proposed epitopes as a vaccine candidate.

**Conclusion:**

Our predicted SARS-CoV-2 epitopes can facilitate vaccine development to enhance the immunogenicity against SARS-CoV-2 and provide supportive data for further experimental validation. Our proposed molecular docking approach of exploiting both MHC and TCR structures can be used to identify potential epitopes for most microbial pathogens, provided the crystal structure of MHC co-crystallized with TCR.

**Supplementary Information:**

The online version contains supplementary material available at 10.1186/s43141-022-00344-1.

## Background

A virus that causes infectious pneumonia broke out at the end of 2019 and rapidly spread worldwide [1]. As it was phylogenetically similar to severe acute respiratory syndrome coronavirus (SARS-CoV) [2], the pathogen has been subsequently identified as a novel coronavirus, SARS-CoV-2 [3], and the associated disease was termed coronavirus disease-19 (COVID-19) [4, 5]. SARS-CoV-2 is more distantly linked to the Middle East respiratory syndrome coronavirus (MERS-CoV) [6], and the T cell responses have been found to give long-term immunity against viral infections [7]. Immune responses by T cells significantly contributed to protection against infection by SARS-CoV, and the pathological damage inflicted by MERS-CoV [8]. The cellular T lymphocyte-mediated responses have been shown to provide the most potent immunity against the structural proteins of SARS-CoV in patients during convalescence [9, 10], as cytotoxic T lymphocytes (CTLs) are known to induce the strongest response to viral infections [11]. Recent studies showed that the development of an epitope-based vaccine can be achieved through recognizing the viral peptides presented by human leukocyte antigens (HLAs) especially peptides of Spike and N proteins [12–14]. During the immune response against the virus, after antigen processing into epitopes through the antigen-presenting cells (APCs), these peptide fragments associate with MHC molecules in a form that is specifically identified by the T cell receptor (TCR).

Furthermore, T cells detect viral antigens presented by MHC class I (the immunogenic peptide–MHC class I complexes), which will enhance CD8+ T cell cytokine production and cytotoxic activity (active effector CTLs) [15]. The alpha-3 domain and beta-2 microglobulin (β2m) of the MHC-I molecule engage with the binding site of the TCR, which consists of two domains arising from a single heavy chain (HC). The two domains combine to form a shallow curved sheet as a base, with the two helices on top, to accommodate a peptide chain “epitope” in-between [16]. The establishment of a set of conserved hydrogen bonds (H bonds) between the side chains of the MHC molecule and the backbone of the peptide is required for binding between the two α-helices and the epitope. The geometry, the hydrophobicity of the binding site, and the charge distribution together determine the type of interactions of peptides with the MHC molecule. Reliable epitope prediction can be achieved through precise prediction of the affinity of the MHC-antigen interactions for individual allotropes [17, 18].

The presentation of a stable immunogenic peptide–MHC class I (MHC) complex is dependent on the fitting between the peptide and the MHC groove, but it is not the only factor. The other factors affecting the formation of MHC complex include protease activity, the accessibility of chaperones, or the antigen. The binding groove of MHC class I is closed on both ends by conserved tyrosine residues, limiting the size of peptides that bind to MHC molecules to roughly 8–10 residues at their C-terminal end docking into the F-pocket [19, 20].

The main objective of our study is to predict the most antigenic SARS-CoV-2 epitopes that are compatible with HLA haplotypes of the Egyptian population. We chose Spike and ORF1ab proteins, as they have a robust scores in several prediction tools including binding prediction with MHC, antigenicity response, and high docking scores with both MHC and TCR. These scores offer significant stability of the provided epitopes, whereas epitope prediction scores measure the affinity between the proposed epitopes and MHC molecules, while antigenicity response measures the ability of the proposed epitopes to elicit an immune response. The scores thus express the stimulation of the immune response against the proposed epitopes. Moreover, molecular docking scores evaluate the most conformational stability of our proposed epitopes with both MHC molecules and T cell receptors. The methods have been selected for their high accuracy in predicting binding conformation and are more fitting with our approach for protein-protein interaction. For example, HDock provides a robust homology modeling strategy for molecular docking via exploiting the FASTA format of the input data instead of the 3D structure prediction molecules. This improves the molecular docking results compared to feeding the 3D structures directly to the docking software. In this case, the software implements different conformation of the predicted epitopes according to their fitting in the binding pocket of both MHC and TCR. Additionally, NetMHCpan4.1 server [21] has a high accuracy score as an epitope prediction platform. The Immune Epitope Database (IEDB) provides a weekly benchmarking with other epitope prediction tools, while NetMHCpan4.1 server has the highest prediction score compared to other tools.

For a more reliable characterization of the epitopes, we used additional tools. Vaxijen [22–24] is a prediction algorithm tool that predicts the antigenic epitopes from three different sources (tumors, bacteria, and virus). The prediction is based on alignment-independent approach, which predicts the antigenicity response relying on the physicochemical properties of the peptides. PEP-FOLD3 [25–27] is a de novo strategy exploiting a linear peptide of amino acid sequence to predict the peptide structure. The structure prediction is relying on a hidden Markov model approach, which has the possibility of creating candidate confirmation by folding the peptides on a set patch of proteins. ToxinPred server [28] was used for toxicity prediction. The server is an in silico method using database of 1805 toxic peptides (≤35 residues). This method is developed to predict and design toxic/non-toxic peptides. AllergenFP v.1.0. server [29] is a bioinformatics tool for allergenicity prediction. This tool is based on a novel descriptor fingerprint approach, which could be applied for any classification problem in computational biology. Finally, ExPASy ProtParam Tool [30] is used for physicochemical properties prediction via computation of various physical and chemical parameters for a given protein. The tool is able to predict the molecular weight, theoretical isoelectric point (pI), amino acid composition, atomic composition, extinction coefficient, estimated half-life, instability index, aliphatic index, and grand average of hydropathicity (GRAVY).

In this study, several SARS-CoV-2 epitopes have been identified using a whole viral protein sequence analysis, exploiting the most updated version of tools for epitope prediction, antigenicity response, and molecular docking. These tools represented the most common and accurate platforms for epitope prediction analysis [21–24]. These proposed epitopes represent the most immunogenic peptides in SARS CoV-2 based on their strong docking affinity with both MHC and TCR. These proposed epitopes have been identified from Spike and ORF1ab proteins for their highest scores in MHC binding affinity, immunogenicity, and molecular docking scores. Due to the genomic variations of the SARS-CoV-2 and HLA haplotypes across populations [31–36], SARS CoV-2 epitopes were identified according to the most common HLA allele frequencies of the Egyptian population [37–39]. Our proposed docking approach of exploiting the structures of both MHC and TCR in validating docking affinity of the proposed epitopes can be applied with any pathogenic protein, as long as the structure of MHC is co-crystallized with TCR.

## Material and methods

### Sequence retrieval and multiple sequence alignment

Genomic sequences of SARS-CoV-2 isolates Egyptian strains [GISAID database [40] (accession ID: EPI_ISL_9047802, EPI_ISL_9047803, EPI_ISL_9047804, EPI_ISL_9047805, EPI_ISL_430820, and EPI_ISL_430819)] [41] were retrieved in FASTA format from GISAID, and a genomic sequence of SARS-CoV-2 isolate Chinese strain [GenBank database [42] (accession ID: NC_045512.2)] [43] was retrieved from GenBank. The viral genomes isolates Egyptian strains were translated into their amino acid sequences using EMBOSS Transeq (https://www.ebi.ac.uk/Tools/st/emboss_transeq), and multiple sequence alignment of amino acid sequences was implemented via ClustalW using Molecular Evolutionary Genetics Analysis software “MegaX” [44, 45].

### Identification of cytotoxic T cell epitopes and their antigenicity response

NetMHCpan4.1 server [21] was exploited to predict viral epitope binding to the most frequent HLA haplotypes in the Egyptian population (HLA-A*0101, HLA-A*0210 HLA-B*03501, HLA-B*4101) [39]. Every SARS-CoV-2 protein was provided to the platform, along with a threshold of 0.5% rank for strong binder and 2 for the weak binder. NetMHCpan4.1 uses artificial neural networks in their predictions, trained on many quantitative binding affinities in addition to mass-spectroscopy eluted ligands. The resulting epitopes were filtered to include only the strong binders with their corresponding HLA haplotypes. Then, antigenicity response was measured by Vaxijen [22–24] for every proposed epitope that was predicted previously. Vaxijen is implemented by using a threshold of 0.4 as a probable antigen. A threshold of 0.4 was selected, as the best prediction threshold of the epitopes’ antigenicity response. Moreover, this score was previously reported to validate the antigenicity response of the proposed epitopes [46–48]. Only crystal structures of HLA-A*0201 and HLA-B*03501 were retrieved from the protein data bank [49–51] under accession ID: 5YXN and 4PRP, respectively.

### Homology modeling

Homology modeling of the resulting probable epitopes was predicted using PEP-FOLD3 [25–27] provided the protein sequences in their FASTA format. Structures with the lowest coarse-grained energy according to PEP-FOLD3 recommendations were selected for molecular docking with MHC-I crystal structures.

### Toxicity and allergenic response

The toxicity and allergenic response of the proposed epitopes were predicted by ToxinPred server (http://crdd.osdd.net/raghava/toxinpred/) [28] and AllergenFP v.1.0. servers (https://ddg-pharmfac.net/AllergenFP/index.html) respectively. Physicochemical properties, including hydropathicity, charge, half-life, instability index, pI (theoretical isoelectric point value), and molecule weight, were predicted by ExPASy ProtParam Tool [30].

### Molecular docking

**Fig. 1:**
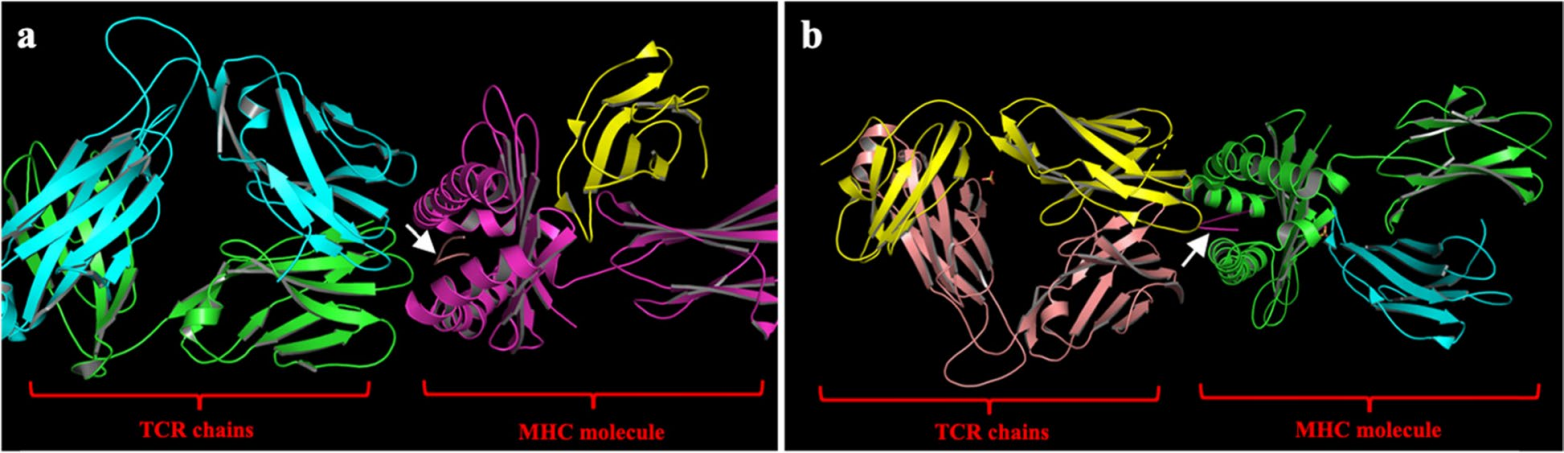
The crystal structures of 5YXN and 4PRP. **a** 5YXN MHC molecule on the right side and TCR chains on the left side. **b** 4PRP MHC molecule on the right side, and TCR chains are on the left side. (white arrows indicate the co-crystalized epitopes)

We adopted the updated version of the HDock server (http://hdock.phys.hust.edu.cn/), which is currently exploited for protein docking based on two methods; template-based and template-free methods, both methods have been exploited to determine the most accurate one in providing high docking scores with both MHC and TCR. We found that template-free method provides more robust docking scores than template-based method. We provide both the crystal structures of MHC-I and TCR chains in PDB format, while the ligands are in their FASTA format. In the molecular docking, we substituted the crystallized epitopes bound between the groove of the MHC and TCR of 5YXN and 4PRP (as shown in brown and pink; Fig. 1a and b, respectively) with our putative SARS-CoV-2 epitopes. The PDB accession ID of MHC crystal structures (5YXN and 4PRP) have been used as input for HDock server along with their interacting chains, chain A for MHC and chains E and D for TCR.

**Fig. 2:**
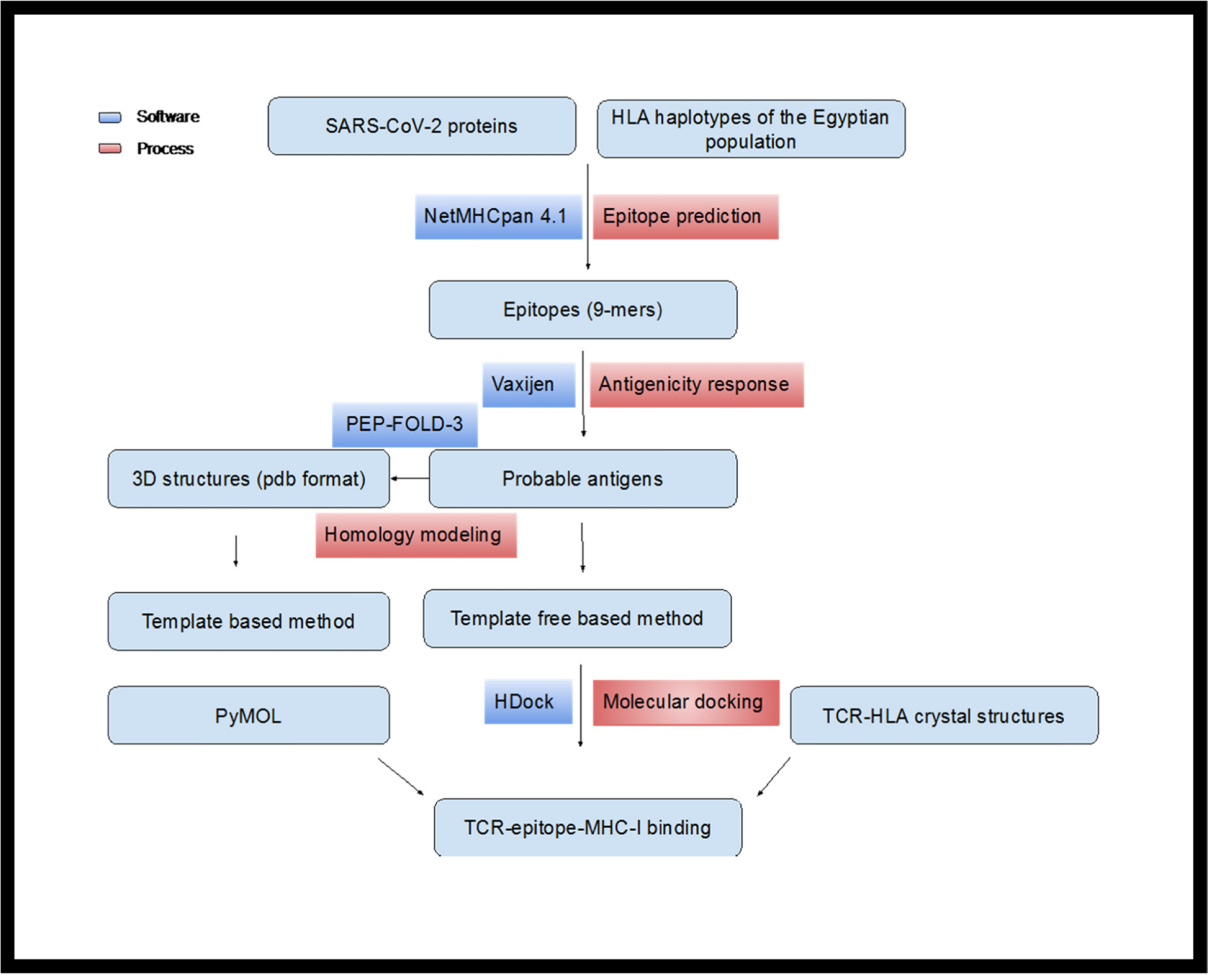
Flow chart of the approach used in epitope prediction of SARS-CoV-2

The interaction of the candidate ligands with their receptors was visualized by PyMOL (https://pymol.org/2/) to investigate the number of interacting bonds between the structures, as depicted in (Fig. 2).

## Results

### Variations in SARS-CoV-2 sequences

**Fig. 3:**
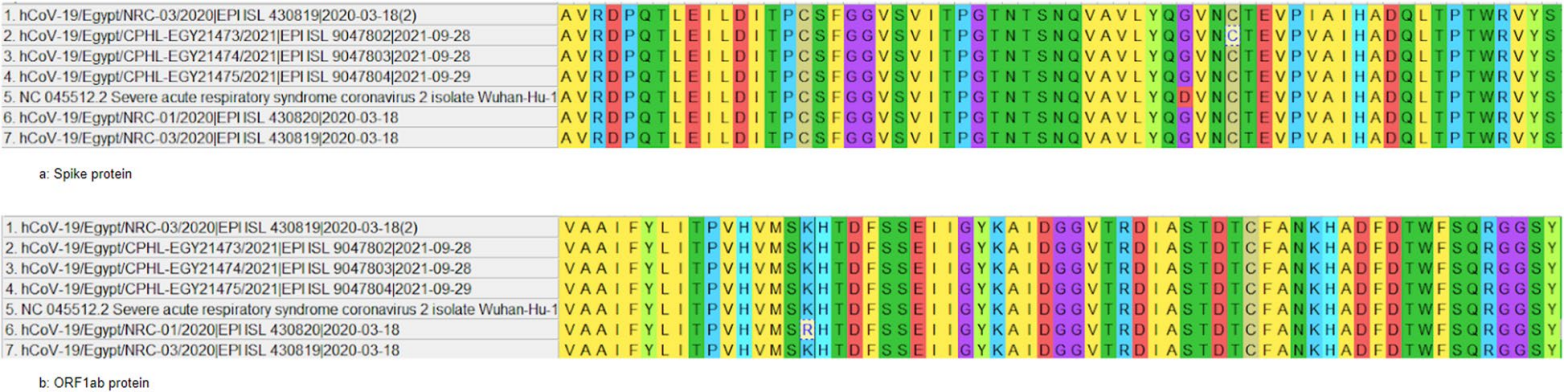
Multiple sequence alignment of both **a** Spike and **b** ORF1ab proteins of SARS-CoV-2 in Wuhan and in Egypt

A number of synonymous mutations between the genomic sequences of SARS-CoV-2 isolated in Wuhan and Egypt were detected. However, two non-synonymous mutations were identified in “Spike” and “ORF1ab” sequences of SARS-CoV-2 in Egypt. The first, presented in all Egyptian strains SARS-CoV-2 isolates, was a mutation of aspartic acid (D) residue at position 7713 to glycine (G) residue in S protein. The second, presented in only one Egyptian strain SARS-CoV-2 isolate was a mutation of lysine (K) residue at position 2798, to arginine (R) in ORF1ab protein (Fig. 3).

### Recognition of CD8^+^ T cell epitopes in SARS-CoV-2

Since Cytotoxic T-lymphocytes recognize certain epitopes attached to MHC-I in the infected cells, T cell epitopes have been identified in our study NetMHCpan4.1 server predicted 406 peptides from all viral proteins, tested with the most common HLA haplotypes of the Egyptian population to evaluate their binding affinity with MHC-I and predict potential CTL epitopes.

### Evaluation of antigenicity and allergenic response

The antigenicity was measured for every epitope by Vaxijen to produce 201 peptides acting as probable antigens (Table 1 and Supplementary Table 1). The Vaxijen score for every epitope provides a robust antigenicity of the proposed epitopes. The allergenicity of the candidate epitopes has been measured by AllergenFP v.1.0. Server (allergenicity scores are listed in Supplementary Table 1). Low allergenic scores indicate that the proposed epitopes might not show any detrimental allergenic reaction.

**Table 1:** The candidate SARS-CoV-2 epitopes for the Egyptian most frequent alleles of MHC class I molecules

Serial in Supplementary Table **1**	Position	HLA haplotype	Epitope sequence	4PRP	5YXN	Antigenicity response	Score	Protein	HLA crystal structure
12	214	HLA-B*35:01, HLA-A*02:01	RDLPQGFSA	− 255.9	− 185.69	0.8947	0.286485	Spike	4PRP, 5YXN
28	269	HLA-B*35:01, HLA-A*02:01	YLQPRTFLL	− 236.05	− 224.82	0.4532	0.972695	Spike	4PRP, 5YXN
25	691	HLA-A*02:01	SIIAYTMSL	− 230.22	− 220.73	0.5234	0.575949	Spike	5YXN
7	2209	HLA-A*02:01	FCLEASFNY	− 314.45	− 191.75	1.5042	0.411451	ORF1ab	5YXN
79	103	HLA-B*35:01, HLA-A*02:01	TLGVLVPHV	− 295.42	− 211.69	0.5583	0.713924	ORF1ab	4PRP, 5YXN
241	3050	HLA-B*35:01	GEYSHVVAF	− 293.81	− 213.74	0.6428	0.523635	ORF1ab	4PRP
14	487	HLA-B*35:01	NCYFPLQSY	− 225	− 183.06	0.8743	0.558006	Spike	4PRP

### Toxicity and physicochemical properties assessment

The toxicity and physicochemical properties of the proposed epitopes were evaluated to validate their quality (Table 2). All of the seven epitope candidates were non-toxic. RDLPQGFSA and NCYFPLQSY epitopes hydrophilic nature and can interact easily with water [52]. The GEYSHVVAF epitope showed the longest half-life of all epitope candidates to be 30 h in vitro and >20 h in vivo. FCLEASFNY, TLGVLVPHV, and GEYSHVVAF epitopes showed instability index < 40, indicating the stable form of these candidates. The GEYSHVVAF epitope shows here the highest stability potential.

**Table 2:** Toxicity and physicochemical properties of the candidate epitopes

Epitopes	Hydropathicity	Half-life (Mammalian reticulocytes, in vitro)	Half-life (Yeast, ***Escherichia coli***, in vivo)	Instability index	Stability	pI	Mol. weight	Toxicity
**RDLPQGFSA**	− 0.656	1 h	2 min, 2 min	51.69	No	5.84	990.08	Non-toxin
**YLQPRTFLL**	0.289	2.8 h	10 min, 2 min	71.84	No	8.75	1150.39	Non-toxin
**SIIAYTMSL**	1.433	1.9 h	>20 h, >10 h	48.28	No	5.24	998.20	Non-toxin
**FCLEASFNY**	0.511	1.1 h	3 min, 2 min	30.29	Yes	4.00	1093.22	Non-toxin
**TLGVLVPHV**	1.589	7.2 h	>20 h, >10 h	30.29	Yes	6.40	934.15	Non-toxin
**GEYSHVVAF**	0.422	30 h	>20 h, >10 h	0.51	Yes	5.24	1008.10	Non-toxin
**NCYFPLQSY**	− 0.322	1.4 h	3 min, >10 h	112.13	No	5.52	1134.27	Non-toxin

### Molecular docking

**Fig. 4:**
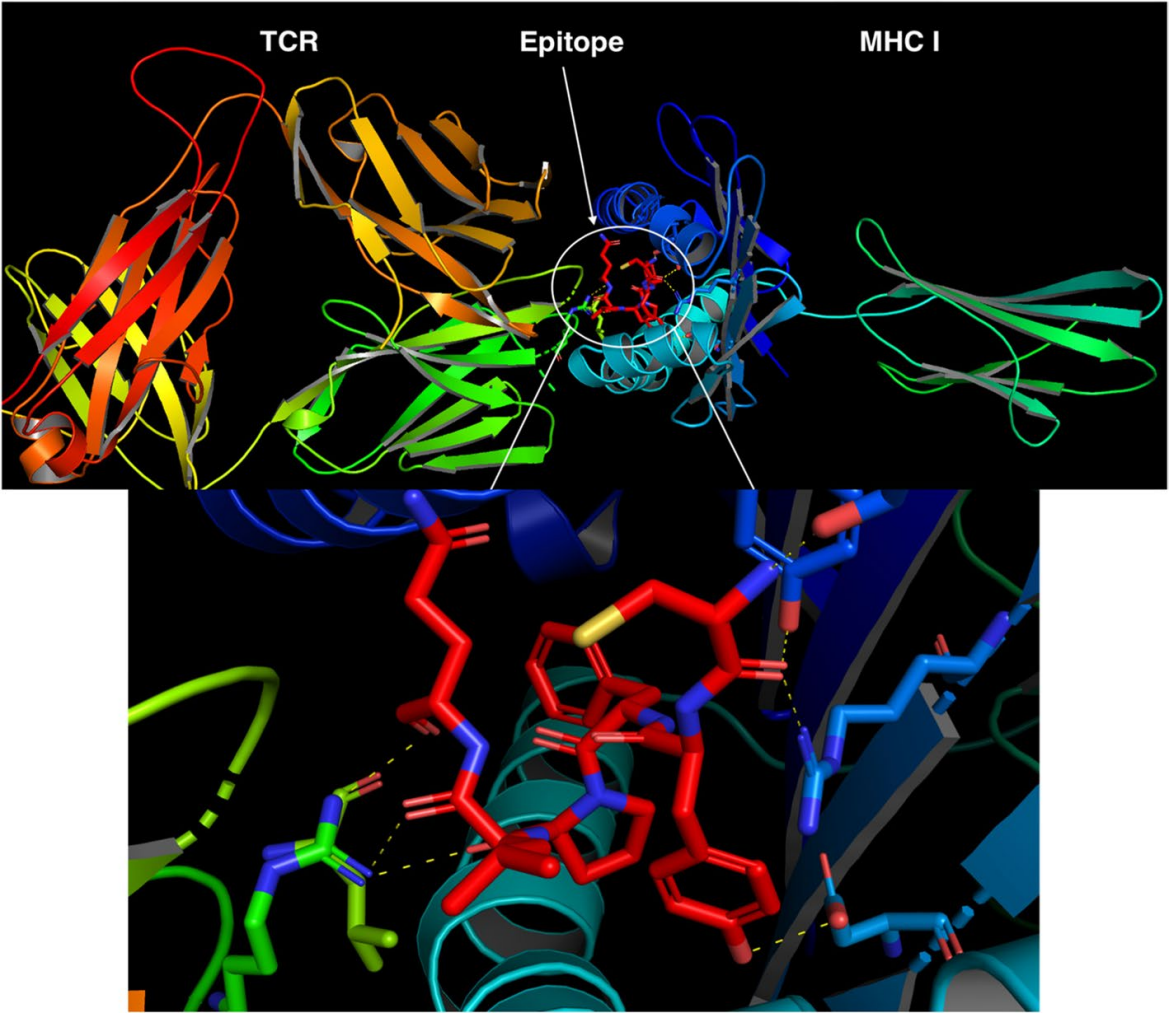
Molecular docking of Spike epitope (No. 14 in Table 1) with both 4prp MHC I molecule and TCR chains

**Fig. 5:**
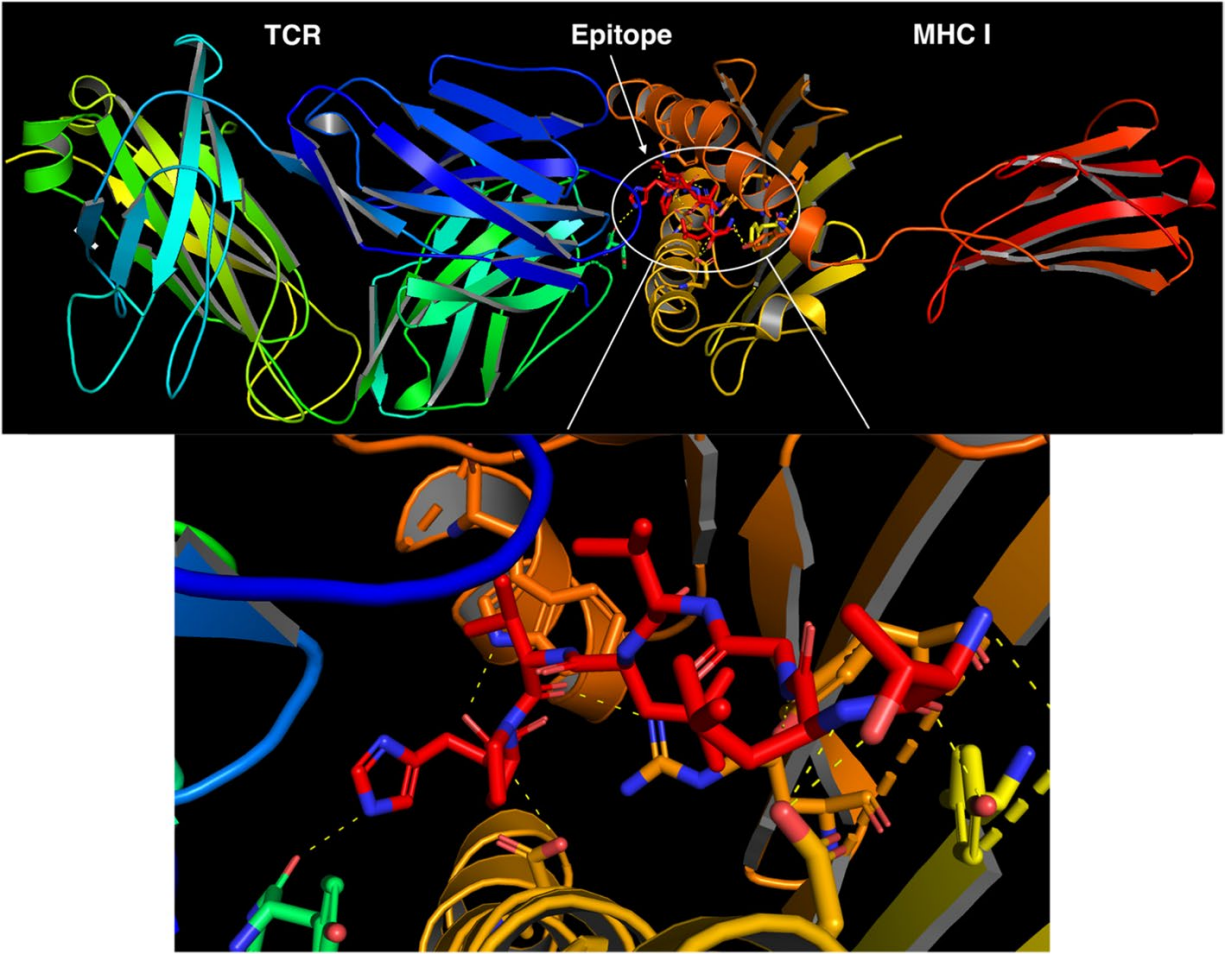
Molecular docking of ORF1ab epitope (No. 79 in Table 1) with both 5yxn MHC I molecule and TCR chains

Molecular docking can evaluate the binding affinity and interaction between the proposed epitope and the target receptor. We obtained several epitopes with high docking scores along the whole viral protein sequences. However, we noticed that the structural Spike and non-structural ORF1ab proteins have the highest docking scores among SARS-CoV-2 proteins (Supplementary Table 1). Ten confirmations for their peptide epitope docking were produced (Supplementary file 1), and top positioned conformations dependent on their docking scores and interactions with MHC-I and TCR residues were visualized to ensure proper binding (Figs. 4 and 5), where they showed the hydrogen bonds (H bonds) that stabilize the candidate epitopes with both MHC class 1 molecule and TCR chains. These H bonds and their bound amino acids along with their bond distances were represented in Table 3. Finally, we found that three of the most promising seven predicted epitopes were shared between both HLA-A 0201 and HLA-B 35:01 (Table 1).

**Table 3:** The amino acids and bond distances between the proposed epitopes and both MHC and TCR

Epitope serial in Supplementary Table **1**	Epitope amino acid [MHC amino acid (distance)]	Epitope amino acid [TCR amino acid (distance)]
14	TYR [ASP (2.6)] CYS [TYR (3.1) CYS [ARG (2.8)] CYS [SER (2.4)]	ARG [LEU (3.3)] ARG [GLN (3.2)] ARG [PRO (2.7)]
241	HIS [ASN (2.9)] PHE [TYR (3.3)] PHE [TYR (3.4)] TYR [ARG (3.3)]	HIS [GLY (3.5)] TYR [ARG (3.2)] TYR [SER (2.4)]
79	THR [GLU (2.5)] LEU [GLU (3.3)] THR [TYR (3.2)] HIS [ASP (3.2)] PRO [TRP (3.2)] VAL [ARG (3.1)] GLY [TYR (3.0)]	HIS [TYR (3.2)]
25	THR [TRP (3.4)] THR [LYS (3.2)] ALA [ARG (3.3)] ILE [TYR (2.7)] ILE [HIS (3.1)]	THR [GLY (2.5)] TYR [TYR (2.3)] THR [ASP (3.3)]
28	TYR [GLU (3.1)] SER [TYR (3.3)] ASN [TYR (2.3)] THR [TYR (3.2)]	TYR [ARG (3.1)] TYR [ASN (3.0)]
12	GLY [LYS (3.4)] GLY [SER (2.3)] GLN [TRP (2.9)] GLN [THR (2.8)]	ARG [ALA (2.8)] ARG [SER (2.4)] ASP [SER (2.7)]

## Discussion

Vaccine development against viral infection is determined by finding the candidate immunogenic epitopes of the viral peptides. Our study aims to determine the putative immunogenic epitopes from the whole viral protein sequence of SARS-CoV-2, which possibly bind to both MHC-I molecule and cytotoxic T cells, as they present the first adaptive line of immune response against viral infection. Epitopes bind to the groove of MHC class I, which is expressed on all nucleated cells. This binding forms a stable conformation leading to antigen presentation and activation of the adaptive immune response CD8^+^ CTLs, which play an indispensable role in combating viral infection [15]. The binding between peptide epitopes and both MHC and TCR is enhanced by the presence of several hydrogen bonds between them, as represented in Table 3 [53, 54]. Due to the polymorphic nature of MHC haplotypes, specific confirmation of peptides can bind with specific MHC molecules [15]. For these variabilities, we sought to predict the candidate epitopes from the whole SARS-CoV-2 viral proteins to precisely determine the best peptide conformation for binding with the corresponding HLA haplotypes of the highest frequency in the Egyptian population [39].

We made several trials for molecular docking by HDock to get the best docking scores, in which we tried both the template-free (FASTA format) and template-based (PDB format) approaches of HDock. We tested both approaches by using the homology modeling structures of the candidate epitopes in their PDB format, which were obtained from the PEP-FOLD3 server, and the epitope protein sequences in FASTA format. We found that the template-free-based model provides higher docking scores than the template-based method. Moreover, by applying our docking approach in providing the alpha and beta chains of TCR, which were co-crystallized with MHC-I molecules, the docking scores and the number of hydrogen bonds increased significantly. This enhanced our analysis and presented a new docking approach by binding the query ligand to both TCR and HLA molecules that stabilize the binding and show a more confident docking conformation.

We located the most favorable vaccine candidates in the Spike and ORF1ab proteins. Similar to other coronaviruses, the Spike protein is a trimeric class I transmembrane glycoprotein located on the surface of SARS-CoV-2 [55]. SARS-CoV-2 Spike protein is involved in receptor recognition, cell attachment, and fusion, making it crucial for viral entry and infectivity [56–61]. On the other hand, ORF1ab has been shown to have key roles in viral interaction with the innate immune response [62, 63], viral replication [64], and viral RNA synthesis and processing [65, 66].

Our study proposed seven immunogenic epitopes, with no toxicity, and with a high antigenicity response that is compatible with their physiochemical properties. Some epitopes are novel and others were predicted in-silico or by experimental techniques [67–70]. The proposed docking approach could provide several antigenic epitopes that were confirmed by several methods experimentally and computationally. CD8^+^ epitope (YLQPRTFLL) has been validated experimentally, which also shows similarity with MERS-CoV epitope for the same HLA haplotype [67, 68]. Another confirmation to our prediction is by re-prediction of other in-silico predicted MHC class I epitope (SIIAYTMSL) that also overlapped with another SARS-CoV-2 MHC class II epitope for DRB1-04:01 and DRB1-07:01. Also, GEYSHVVAF, NCYFPLQSY, and TLGVLVPHV were previously predicted to different HLA haplotype binding [69, 70]. We however predicted the immunogenic potential of all epitopes by docking with both MHC-I and TCR chains. The data are in agreement with other studies that suggested some of these epitopes as potential targets for vaccine development [71–73]. Additionally, we have predicted other novel SARS-CoV-2 immunogenic epitopes. Experimental validation of these candidates is promising for both therapeutic applications and vaccine development.

The exploited HLA haplotypes represented the highest frequencies in the Egyptian population and also in worldwide population (HLA-A*01:01 16.2%, HLA-A*02:01 25.2%, HLA-B*35:01 6.5%) [70]. The predicted epitopes thus not only fit with the HLA haplotypes of the Egyptian population but can be also applied worldwide. Despite the highest docking scores and MHC binding affinity of the putative epitopes, in-vitro experimental validation or in vivo studies are required to confirm their immunogenicity against SARS-CoV-2.

## Conclusion

We identified seven SARS-CoV-2 epitopes from Spike and ORF1ab proteins, according to the most common HLA allele frequencies of the Egyptian population. Some of these epitopes were previously validated in vitro and in silico and others are novel SARS-CoV-2 epitopes, characterized by a high probability of eliciting an immune response and stable molecular interaction. This was indicated by the high antigenicity, highest docking scores, and docking stability of these epitopes with both MHC class I and TCR chains that were stabilized by several hydrogen bonds. Importantly, our molecular docking approach is more feasible and useful when using the structure of MHC molecules co-crystallized with their TCR chains, and not only using the crystal structure of MHC molecules as followed in many recent studies. This molecular docking approach of utilizing both MHC and TCR structures for epitope prediction can be extended to most microbial infections. Experimental validation of these proposed epitopes should ultimately confirm their binding and interaction with specific TCRs, immunogenic response, and therapeutic potential against SARS-CoV-2.

## Supplementary Information

**Additional file 1: Figure S1.** Molecular docking of (a, b) ORF1ab and (c – e) Spike epitopes (No. 17, 12, 25 & 29 in the Supplementary Table 1) with both 5YXN MHC I molecules and TCR chains. **Figure S2.** Molecular docking of (a, b) ORF1ab and (c – e) Spike epitopes (No. 24, 79, 12, & 29 in Supplementary Table 1) with both 4PRP MHC I molecule and TCR chains.

**Additional file 2: Supplementary Table 1.**

## Data Availability

All data generated or analyzed during this study are included in this published article and its supplementary information files.

## Abbreviations

SARS: Severe acute respiratory syndrome
CTL: Cytotoxic T lymphocyte
HLA: Human leukocyte antigen
MHC: Major histocompatibility molecules
