# Correction: Immunoinformatics approach of epitope prediction for SARS-CoV-2

**DOI:** 10.1186/s43141-022-00350-3

**Published:** 2022-05-09

**Authors:** Nourelislam Awad, Rania Hassan Mohamed, Nehal I. Ghoneim, Ahmed O. Elmehrath, Nagwa El-Badri

**Affiliations:** 1 Center of Excellence for Stem Cells and Regenerative Medicine (CESC), Zewail City of Science and Technology, Giza, Egypt; 2 Center of Informatics Sciences, Nile University, Giza, Egypt; 3 Department of Biochemistry, Faculty of Science, Ain Shams University, Cairo, Egypt; 4 Faculty of Medicine, Cairo University, Cairo, Egypt

**Correction: J Genet Eng Biotechnol 20, 60 (2022)**

**https://doi.org/10.1186/s43141-022-00344-1**

Following publication of the original article [1], the authors identified an error in the HTML version of Fig. 1. The publisher apologise for this error. The correct figure is given hereafter.

**Fig. 1:**
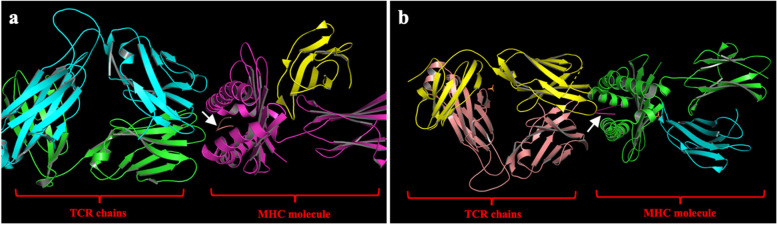
The crystal structures of 5YXN and 4PRP. **a** 5YXN MHC molecule on the right side and TCR chains on the left side. **b** 4PRP MHC molecule on the right side, and TCR chains are on the left side. (white arrows indicate the co-crystalized epitopes)

The original article [1] has been corrected.
