# Supplementary material for: Immunoinformatics approach of epitope prediction for SARS-CoV-2
Source: J Genet Eng Biotechnol. 2022 Apr 20;20:60. doi: 10.1186/s43141-022-00344-1 (PMC9019534; doi:10.1186/s43141-022-00344-1)
Supplement: Supplementary file 1 — Additional file 1: Figure S1. Molecular docking of (a, b) ORF1ab and (c – e) Spike epitopes (No. 17, 12, 25 & 29 in the Supplementary Table 1) with both 5YXN MHC I molecules and TCR chains. Figure S2. Molecular docking of (a, b) ORF1ab and (c – e) Spike epitopes (No. 24, 79, 12, & 29 in Supplementary Table 1) with both 4PRP MHC I molecule and TCR chains. [file 43141_2022_344_MOESM1_ESM.pdf]

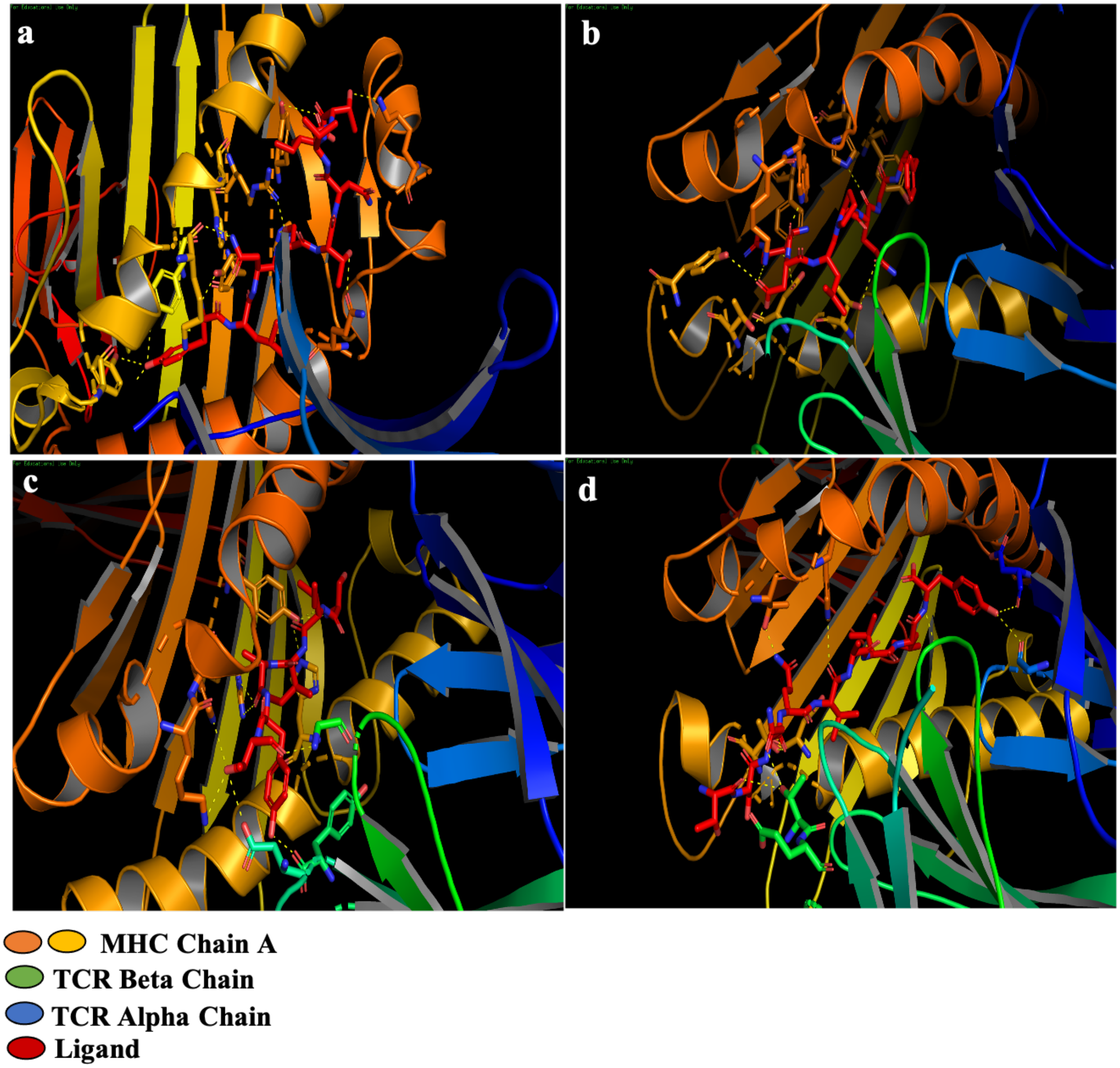

Figure S1. Molecular docking of (a, b) ORF1ab and (c – e) Spike epitopes (No. 17, 12, 25 & 29 in the Supplementary Table) with both 5YXN MHC I molecules and TCR chains.

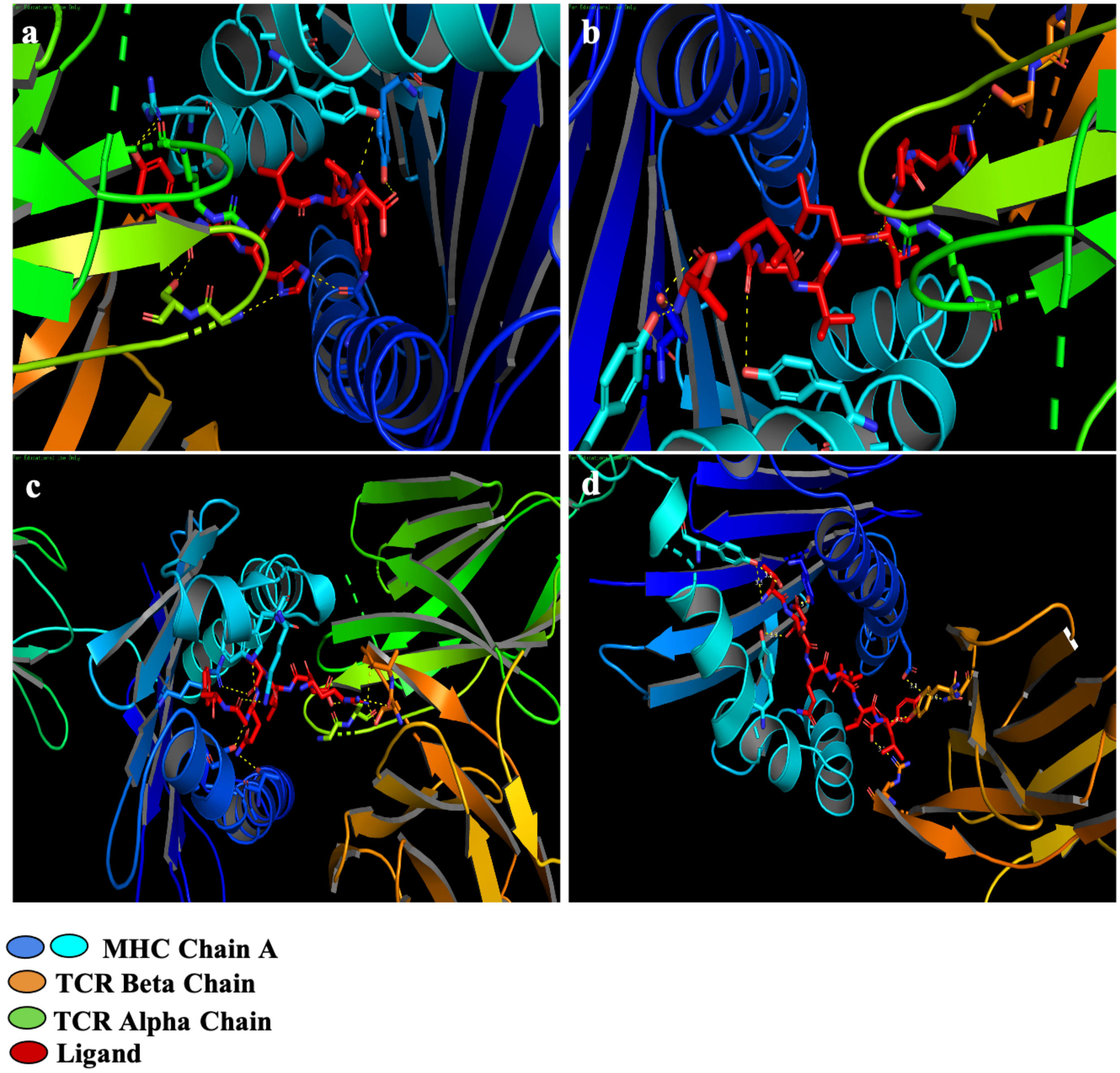

Figure S2. Molecular docking of (a, b) ORF1ab and (c – e) Spike epitopes (No. 24, 79, 12, & 29 in Supplementary Table) with both 4PRP MHC I molecule and TCR chains
